# Mechanism Profiling of Hepatotoxicity Caused by Oxidative Stress Using Antioxidant Response Element Reporter Gene Assay Models and Big Data

**DOI:** 10.1289/ehp.1509763

**Published:** 2015-09-18

**Authors:** Marlene Thai Kim, Ruili Huang, Alexander Sedykh, Wenyi Wang, Menghang Xia, Hao Zhu

**Affiliations:** 1 Department of Chemistry, Rutgers University, Camden, New Jersey, USA; 2 Center for Computational and Integrative Biology, Rutgers University, Camden, New Jersey, USA; 3 National Center for Advancing Translational Sciences, National Institutes of Health, Department of Health and Human Services, Bethesda, Maryland, USA; 4 Multicase Inc., Beachwood, Ohio, USA

## Abstract

**Background:**

Hepatotoxicity accounts for a substantial number of drugs being withdrawn from the market. Using traditional animal models to detect hepatotoxicity is expensive and time-consuming. Alternative in vitro methods, in particular cell-based high-throughput screening (HTS) studies, have provided the research community with a large amount of data from toxicity assays. Among the various assays used to screen potential toxicants is the antioxidant response element beta lactamase reporter gene assay (ARE-bla), which identifies chemicals that have the potential to induce oxidative stress and was used to test > 10,000 compounds from the Tox21 program.

**Objective:**

The ARE-bla computational model and HTS data from a big data source (PubChem) were used to profile environmental and pharmaceutical compounds with hepatotoxicity data.

**Methods:**

Quantitative structure–activity relationship (QSAR) models were developed based on ARE-bla data. The models predicted the potential oxidative stress response for known liver toxicants when no ARE-bla data were available. Liver toxicants were used as probe compounds to search PubChem Bioassay and generate a response profile, which contained thousands of bioassays (> 10 million data points). By ranking the in vitro–in vivo correlations (IVIVCs), the most relevant bioassay(s) related to hepatotoxicity were identified.

**Results:**

The liver toxicants profile contained the ARE-bla and relevant PubChem assays. Potential toxicophores for well-known toxicants were created by identifying chemical features that existed only in compounds with high IVIVCs.

**Conclusion:**

Profiling chemical IVIVCs created an opportunity to fully explore the source-to-outcome continuum of modern experimental toxicology using cheminformatics approaches and big data sources.

**Citation:**

Kim MT, Huang R, Sedykh A, Wang W, Xia M, Zhu H. 2016. Mechanism profiling of hepatotoxicity caused by oxidative stress using antioxidant response element reporter gene assay models and big data. Environ Health Perspect 124:634–641; http://dx.doi.org/10.1289/ehp.1509763

## Introduction

Using traditional animal models to evaluate hepatotoxicity is expensive and time-consuming (Hartung 2009). *In vitro* assays are used as an alternative to increase our understanding of hepatotoxicity (Adler et al. 2011; Zhu et al. 2013). However, endeavors to correlate *in vitro* and *in vivo* hepatotoxicity (Moeller 2010) have not successfully replaced *in vivo* hepatotoxicity models (Ekins 2014; MacDonald and Robertson 2009).

There is an unmet need to develop predictive assays for hepatotoxicity (Chen et al. 2014). As an alternative, high-throughput screening (HTS) approaches are used to screen large chemical libraries (> 50,000 compounds) to elucidate toxic mechanisms and to prioritize candidate compounds for further animal testing (Zhu et al. 2014). This approach leads to the rapid generation of bioassay data. PubChem, the leading public bioassay data repository, contains > 50 million compounds and > 700,000 assays (Wang et al. 2014). This amount of “big data” is difficult to process and analyze using standard data-processing tools.

Another disadvantage of using HTS for toxicological studies is that this method tests compounds at only one concentration, which may not reveal its toxic effects. This problem was addressed by the U.S. Tox21 interagency collaboration [Attene-Ramos et al. 2013; Collins et al. 2008; National Research Council (NRC) 2007; Dix et al. 2007]. Based on their guidelines, the National Institutes of Health Chemical Genomics Center (NCGC), now part of the National Center for Advancing Translational Sciences (NCATS), developed quantitative high-throughput screening (qHTS) (Inglese et al. 2006). A qHTS experiment tests > 100,000 compounds at 15 different concentrations in triplicate within one week (Attene-Ramos et al. 2013). This approach is more rational than single-dose HTS because it simulates dose-dependent animal toxicity effects (Eaton and Gilbert 2010). These results are available online (NCBI PubChem BioAssay, search term “tox21;” http://www.ncbi.nlm.nih.gov/pcassay?term=tox21; accessed 19 January 2015).

The antioxidant response element (ARE) pathway plays a major role in regulating and alleviating oxidative stress (Ma 2013), which after long-term exposure causes many pathophysiological conditions, including cancers and hepatotoxicity (Hybertson et al. 2011; Shuhendler et al. 2014). Briefly, the ARE pathway is regulated by Kelch-like ECH-associated protein 1 (Keap1) and nuclear factor erythroid 2-related factor 2 (Nrf2). Keap1 contains cysteine residues that interact with reactive oxygen species (ROS) and electrophilic fragments that can trigger the dissociation of the Keap1-Nrf2 complex (Zhang and Hannink 2003). Then, Nrf2 translocates into the nucleus (Kensler et al. 2007), binds to the ARE (Itoh et al. 1997), and regulates the transcription of antioxidative enzymes (Venugopal and Jaiswal 1998). Hindering antioxidant transcription can lead to the accumulation of ROS, oxidative stress, and liver toxicity (Shuhendler et al. 2014). The qHTS ARE *beta* lactamase reporter gene assay (ARE-*bla*) can detect compounds that activate the ARE pathway and induce oxidative stress (Attene-Ramos et al. 2013; Shukla et al. 2012; Simmons et al. 2011). However, this assay alone is not sufficient for assessing animal toxicity. The correlations between the ARE pathway and animal toxicity (i.e., hepatotoxicity) are not well understood.

Despite the substantial data obtained from HTS and/or qHTS studies, the relationship between *in vitro* and *in vivo* toxicity remains unclear (Low et al. 2011; O’Brien et al. 2006). In the present study, this challenge was addressed by developing chemical *in vitro–in vivo* correlations (IVIVCs) between ARE pathway activation and hepatotoxicity (i.e., liver damage). An in-house automated profiling tool used qHTS ARE-*bla* and liver toxicity data to retrieve relevant assays from PubChem and revealed liver toxicity targets. Analyzing chemical fragments of liver toxicants revealed potential toxicophores (toxic chemical features) with clear IVIVCs for a subset of compounds. Our study suggests that the use of assays as an alternative model for toxicity is feasible based on chemical IVIVCs identified from a big data source.

## Methods

*Databases.* qHTS ARE-*bla* data set. The initial concentration–response profiles for the Tox21 10K collection tested in the qHTS ARE-*bla* tests were conducted at the NCATS (Attene-Ramos et al. 2013; Shukla et al. 2012). The Tox21 10K chemical library [U.S. Environmental Protection Agency (EPA) 2012] contains compounds procured from commercial sources by the U.S. EPA, the National Toxicology Program (NTP), and the NCGC (Huang et al. 2011) for a total of ~10,500 plated compound solutions consisting of 8,311 unique chemical substances including pesticides, industrial and food-use compounds, and drugs. The qHTS ARE-*bla* data sets can also be downloaded from PubChem using Bioassay Accession Identifiers (AIDs) 743219 and 651741. PubChem is a public repository for chemical structures and their biological properties (Wang et al. 2014). Bioactivity data in PubChem are contributed by hundreds of institutes, research laboratories, and specifically by screening centers under the NIH Molecular Libraries Program (MLP) and the Tox21 program. Descriptions of the individual datasets are listed in Table 1.

**Table 1:** Comprehensive toxicity databases compiled from public sources.

Name	Type	Description	Number of compounds
Tox21 Phase I (NTP and EPA) ARE-*bla* [National Center for Biotechnology Information (NCBI) 2015]	*In vitro*	Compounds characterized in traditional toxicology tests and/or known to be harmful to humans and the environment	2,617
Tox21 Phase II 10K ARE-*bla* (U.S. EPA 2012)	*In vitro*	Diverse compounds (pesticides, industrial and food-use compounds, drugs, etc.) with chemical features that are of interest to toxicologists	8,311
FDA liver damage (Zhu and Kruhlak 2014)	*In vivo*	Drugs known to cause liver damage (e.g., necrosis, lesions, traumatic liver injury)	1,314
PubChem Bioassay (NCBI 2014)	*In vitro* and *in vivo*	Compounds that have been validated and screened in different bioassays	> 48,000,000

The concentration–response data were normalized, range-scaled to [0, 100], and converted into curve fingerprints (Sedykh et al. 2011) using an in-house program. The source code can be downloaded from GitHub (https://github.com/sedykh/curvep). Each curve fingerprint was summed into one value termed “CurveP.” CurveP represents the overall signal of the compound from its noise-filtered qHTS concentration–response curve (e.g., CurveP = 0 means no significant signals observed). Three criteria were used to classify each compound with regard to activity: *a*) CurveP, *b*) maximum concentration–response, and *c*) number of concentration–responses ≥ 20. The last two criteria describe the consistency of the concentration–responses. The scheme is detailed in Table 2. For example, a compound was classified as active if CurveP was > 0 and more than one concentration–response was ≥ 20. Lastly, because all compounds were tested multiple times and because all data were available, the activities of each compound were averaged before classification.

**Table 2:** Definition of compound activity categories from concentration–response curves and the CurveP algorithm for the qHTS ARE-*bla* datasets.

Category	Activity	CurveP	Maximum response	Number of responses > 20 units
Active^*a*^	1	> 0	≥ 20	> 1
Potential active^*b*^	0.75	> 0	≥ 20	= 1
Inconclusive^*c*^	0.25	= 0	< 20	= 0
Inactive^*d*^	0	= 0	< 10	= 0
qHTS ARE-*bla*, Quantitative high-throughput screening antioxidant response element *beta* lactamase reporter gene assay.^***a***^Strong ARE-*bla* activation signals observed. ^***b***^Weak ARE-*bla* activation signals observed. ^***c***^Inconsistent ARE-*bla* activation signals observed. ^***d***^Negligible or no ARE-*bla* activation signals observed.				

*In vivo* hepatotoxicity data set. A liver damage data set compiled by the U.S. Food and Drug Administration (FDA) Center for Drug Evaluation and Research (Zhu and Kruhlak 2014) and Multicase Inc. contained 1,314 compounds (661 toxic and 653 nontoxic).

*Chemical structure curation.* The structures of all compounds used in the present study were curated to remove errors and standardized to a uniform representation. Konstanz Information Miner (KNIME) v.2.9.2 (KNIME.com AG, Zurich, Switzerland) matched all compound names and PubChem Compound Accession Identifiers (CIDs) with their appropriate simplified molecular-input line-entry system (SMILES) formulas from PubChem. The in-house descriptor generators could not process large molecules (molecular weight > 2,000 g/mol) or compounds without available chemical structures. These compounds were removed from our data set. ChemAxon Standardizer and Structure Checker v.6.2.2 (ChemAxon, Budapest, Hungary) and CASE Ultra v.1.5.0.1 (MulitCASE Inc., Beachwood, OH) curated, standardized, and converted all the chemical structures into 2-D SMILES representations. Stereoisomers were considered as one compound. Metalorganics were removed and all salts were neutralized because the descriptor generator cannot process them. Mixtures were manually evaluated, and the major component was retained.

*Measures of quality and reliability.* To systematically evaluate the quality and reliability of the quantitative structure–activity relationship (QSAR) models and IVIVCs developed in this study, we calculated the sensitivity and specificity of each assay relative to *in vivo* animal toxicity data and derived the correct classification rate (CCR) where CCR = [(sensitivity + specificity)/2] × 100 (Daniel 2009; Kim et al. 2014). In addition, we calculated the likelihood parameter (*L*) as an indication of the likelihood that active responses in a bioassay correlated with *in vivo* toxicity outcomes, where *L* = sensitivity × [(false positives + true positives)/(false positives + 1)] (Zhang et al. 2014). The statistical significance of the IVIVCs was determined using chi-squared (*χ^2^*) tests comparing the *in vitro* assay predictions with expectations based on *in vivo* toxicity data under the null hypothesis of no association between the two data sources (Daniel 2009).

**Figure 1:**
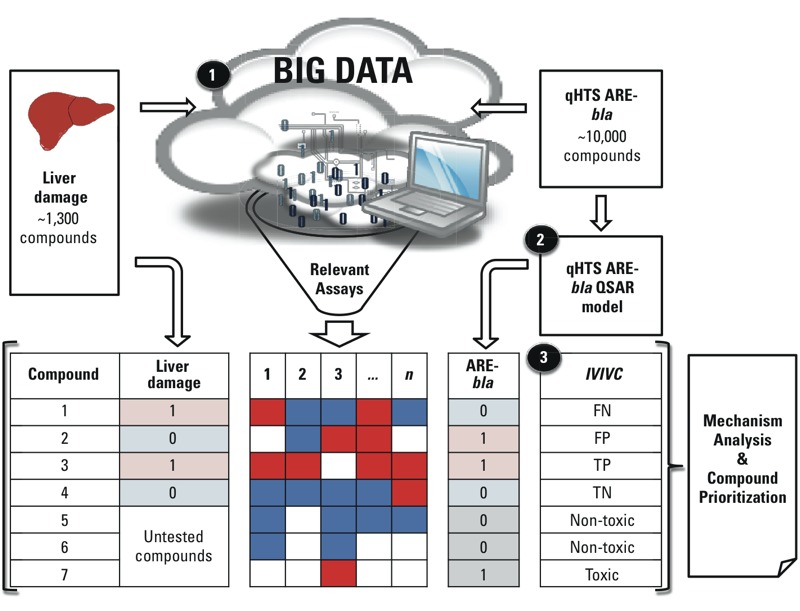
The workflow for profiling liver toxicants consists of three major stages: (*1*) automated biological response profiling, (*2*) quantitative structure–activity relationship (QSAR) modeling of quantitative high-throughput screening antioxidant response element *beta* lactamase reporter gene (qHTS ARE-*bla*) activation, and (*3*) chemical IVIVC evaluation. In the columns (Liver Damage, 1, 2, 3, “…”, *n,* ARE-*bla*), actives are red and “1;” inactives are blue and “0;” and inconclusive or untested are white and empty.

*Workflow for profiling the mechanisms of liver toxicants.* The chemical IVIVC between qHTS ARE-*bla* perturbation or relevant PubChem assays and liver damage was evaluated. The profiling workflow has three major stages (Figure 1): *a*) automated biological response profiling, *b*) QSAR modeling of qHTS ARE-*bla* activation, and *c*) chemical IVIVC evaluation.

*Automated biological response profiling.* The biological response profile was constructed from PubChem Bioassay data (NCBI 2014) with an in-house automated profiling tool (Zhang et al. 2014), which resulted in two profile groups. One group was related to qHTS ARE-*bla* activation, and the second was related to liver damage. The correlations between all bioassays (> 2,000) and ARE-*bla* and liver damage were calculated (sensitivity, specificity, CCR, and *L*). Only bioassays that fit the following criteria were considered for the final biological response profile: *a*) appearance in both profile groups; *b*) > 10 active responses that matched the inputted data; *c*) better than random correlation (CCR > 0.5 and *L* ≥ 1); and *d*) *in vitro* assay. Lastly, bioassays were selected for further analysis if there was evidence in the literature showing that these assays were used to study oxidative stress and/or liver damage.

It was hypothesized that compounds that were active in multiple assays but were not pan assay interference compounds (Baell and Holloway 2010) (i.e., compounds showing false positive results in many assays because of assay technology–specific artifacts) were more likely to be toxic. Using the responses from the selected assays, the rate of actives (RA) was calculated to represent all of the bioassay responses for each compound:

[1]
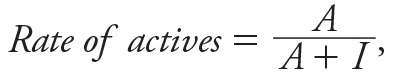


where *A* is the number of active responses, and *I* is the number of inactive responses for a compound. The RA parameter was designed for big data research because missing data can occur in the response profiles for target compounds. For example, if four assays were identified and a compound tested in all four assays was active in one assay and negative in the other three assays, the compound would have an RA = 0.25. However, if another compound was active in one assay, negative in two assays, and produced no data or an inconclusive result for the fourth assay, it would have an RA = 0.33. Thus, potential bias caused by missing assay data can be reduced. An arbitrary RA threshold was used to distinguish toxic compounds from nontoxic compounds (RA > 0.25 as toxic, RA ≤ 0.25 as nontoxic). The RA values were used to determine the IVIVC between liver damage and the assays. To measure the quality and reliability of the assays, each RA value was classified as true positive (TP), true negative (TN), false positive (FP), or false negative (FN) for a *χ^2^* test (α = 0.05).

*QSAR modeling of the ARE-*bla *pathway.* The qHTS ARE-*bla* data sets were used to develop qHTS ARE-*bla* combinatorial QSAR models. Two-dimensional chemical descriptors for each compound were generated using Molecular Operating Environment (MOE) v.2011.10 (Chemical Computing Group Inc., Montreal, Canada) and Dragon v.6.0. (Talete s.r.l., Milano, Italy). All descriptors were normalized and range scaled to [0, 1]. In total, 186 MOE and 2,629 Dragon descriptors were used to model qHTS ARE-*bla* activation.

The qHTS ARE-*bla* data set was down-sampled using a chemical similarity search approach to balance the ratio of active and inactive compounds selected for modeling (Sedykh et al. 2011; Willett et al. 1998). This approach prevents the development of biased models. Active and inactive compounds from the Tox21 Phase II data set were selected to create the modeling set because it was much larger than the Tox21 Phase I data set (Golbraikh et al. 2003; Tice et al. 2013). A principal component analysis was performed using all 186 MOE descriptors. Individual models were developed using combinations of MOE or Dragon descriptors and random forest (RF) (Breiman 2001), support vector machine (SVM) (Vapnik 2000), or *k*-nearest neighbor (*k*-NN) (Zheng and Tropsha 2000) algorithms. Six different combinations of descriptors and algorithms were used for modeling: MOE-RF, MOE-SVM, MOE-*k-*NN, Dragon-RF, Dragon-SVM, and Dragon-*k-*NN. Modeling results were averaged into a consensus model. Models were validated using 5-fold external cross-validation (80/20% split). Additional details about QSAR modeling and validation approaches can be found elsewhere (Golbraikh et al. 2003; Kim et al. 2014; Tropsha and Golbraikh 2007).

Because prediction values ranged from [0, 1], two consensus prediction thresholds (CPTs) (Kim et al. 2014) were defined to classify compounds as active or inactive: CPT-1 (≥ 0.5 as active and < 0.5 as inactive), and CPT-2 (≥ 0.8 as active and ≤ 0.3 as inactive). Predictions between CPT-2 thresholds (< 0.8 and > 0.3) were inconclusive. An applicability domain (AD) determined whether the external compounds were structurally dissimilar to the modeling set compounds (Tropsha and Golbraikh 2007). Predictions of compounds outside the AD were considered unreliable. Therefore, the coverage (fraction of compounds within the AD) was calculated when applying the AD to the predictions.

*Chemical IVIVC evaluation.* Potential toxicophores, chemical fragments with significant IVIVCs, were identified by inputting compounds active in the qHTS ARE-*bla* and liver damage data sets into CASE Ultra and ChemoTyper version 1.0. The substructure search tool in KNIME searched the qHTS ARE-*bla* and liver damage data sets for compounds containing the potential toxicophores. The qHTS ARE-*bla* combinatorial QSAR models predicted compounds from the liver damage data set that were not tested in the qHTS ARE-*bla* assay. The predictions were classified as TP, TN, FP, or FN to evaluate the chemical IVIVC for each subset of compounds with potential toxicophores. The chemical IVIVC results were determined using sensitivity, specificity, CCR, and *χ^2^* analyses (α = 0.05) (Daniel 2009).

## Results

*Overview of qHTS ARE-*bla *data set.* The original qHTS ARE-*bla* data contained two data sets (Tox21 Phase I and Phase II). After combining, curating, and standardizing the chemical structures and activities, 6,767 unique compounds (919 actives, 748 potential actives, 760 inconclusives, and 4,340 inactives) remained. Potential active and inconclusive compounds were excluded from further analyses. The remaining Phase I data set consisted of 1,474 unique compounds (341 actives and 1,133 inactives), and the Phase II data set consisted of 5,134 unique compounds (878 actives and 4,256 inactives).

**Figure 2:**
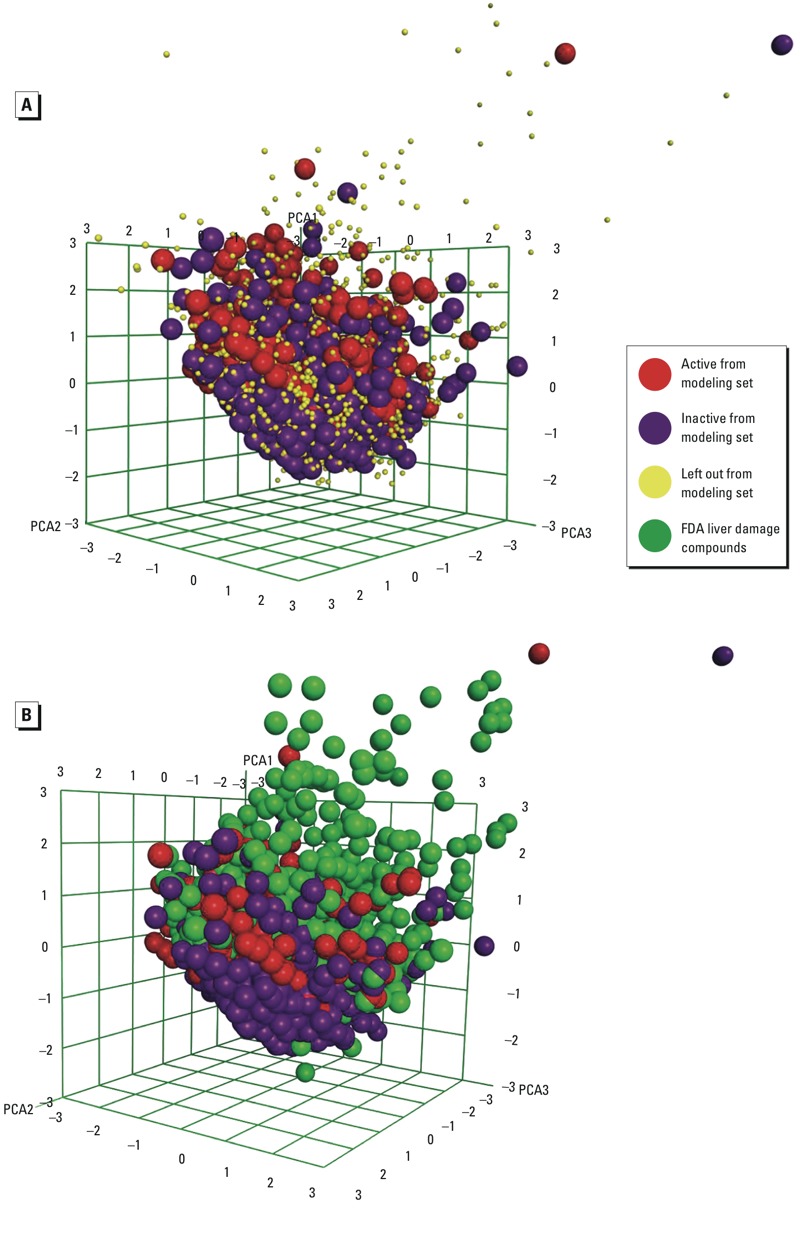
Chemical space plot of (*A*) the modeling set (actives = red, inactives = purple) versus left out compounds (yellow) and (*B*) the modeling set versus the FDA liver damage compounds (green) using the top three principal components generated using 186 MOE 2-D descriptors.

*qHTS ARE-*bla *combinatorial QSAR models.* Seven qHTS ARE-*bla* QSAR models were developed for the modeling set (six individual models and one consensus model). The down-sampled modeling set contained 1,550 (750 actives and 800 inactives) unique compounds. Compounds left out of the modeling sets were placed into external validation sets. Three-dimensional chemical space plots of the modeling set versus its left-out compounds and versus the liver damage data set are shown in Figure 2A and 2B, respectively. External validation sets I (from Tox21 Phase I) and II (from Tox21 Phase II) contained 1,148 (175 active and 973 inactive) and 3,584 (128 active and 3,456 inactive) compounds, respectively. The predictions of these QSAR models for new compounds represent the potential effects of these chemicals (either activation or no effect) in the qHTS ARE-*bla*.

The performance of the qHTS ARE-*bla* combinatorial QSAR consensus model in the 5-fold cross-validation and against the external validation sets, with an AD for CPTs 1 and 2, are shown in Table 3. The consensus modeling set showed good performance in the 5-fold cross-validation (sensitivity = 75–76%, specificity = 71–92%, and CCR = 74–84%). The performance of the consensus model against external validation sets I and II without an AD was satisfactory (sensitivity = 68–93%, specificity = 72–99%, and CCR = 77–92%). When an AD was used, the performance of the external validation sets continued to be acceptable (sensitivity = 62–90%, specificity = 78–99%, CCR = 79–93%, coverage = 34–77%). The individual models showed acceptable performance in the 5-fold cross validation (sensitivity = 68–77%, specificity = 58–73%, and CCR = 67–73%) (see Supplemental Material, Figure S1). Overall, the consensus prediction results were comparable to the results of the best individual model, Dragon-RF (sensitivity = 74%, specificity = 73%, CCR = 73%) (see Supplemental Material, Figure S1).

**Table 3:** qHTS ARE-*bla* combinatorial QSAR consensus model performance in 5-fold cross validation and against external validation sets, with and without applicability domain consensus prediction thresholds 1–2.

Statistic	5-fold cross-validation(80/20% split)	Validationset I	Validation set I + AD	Validation set II	Validation set II + AD
*n* (active/inactive)	750/800	175/973	132/757	128/3,456	59/2,566
CPT-1^*a*^
Sensitivity^*c*^ (%)	76	76	73	83	80
Specificity^*d*^ (%)	71	83	85	72	78
CCR (%)	74	80	79	77	79
Coverage^*e*^ (%)	100	100	77	100	73
CPT-2^*b*^
Sensitivity^*c*^ (%)	75	68	62	93	90
Specificity^*d*^ (%)	92	99	99	92	95
CCR (%)	84	84	80	92	93
Coverage^*e*^ (%)	35	40	34	45	37
Abbreviations: AD, applicability domain; CCR, correct classification rate; CPT, consensus prediction threshold; qHTS ARE-*bla*, Quantitative high-throughput screening antioxidant response element *beta* lactamase reporter gene assay; QSAR, quantitative structure–activity relationship^***a***^CPT-1: QSAR prediction ≥ 0.5 as actives and QSAR prediction < 0.5 as inactives. ^***b***^CPT-2: QSAR prediction ≥ 0.8 as actives and QSAR prediction ≤ 0.3 as inactives. ^***c***^Percentage of active or toxic compounds predicted correctly. ^***d***^Percentage of inactive or nontoxic compounds predicted correctly. ^***e***^Fraction of compounds within the applicability domain.					

**Figure 3:**
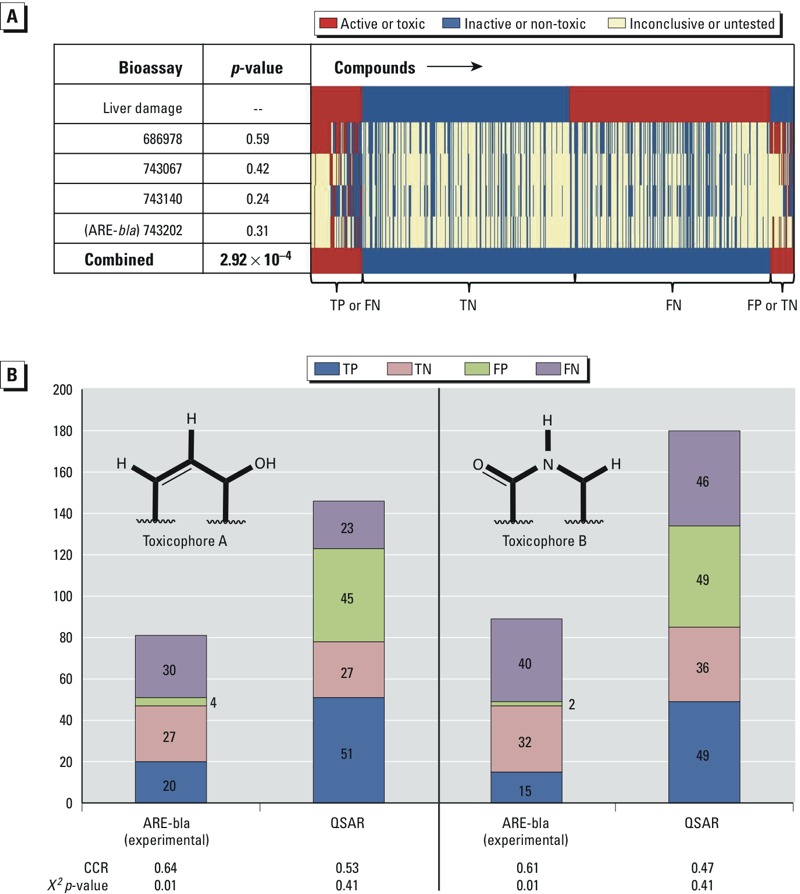
The IVIVC between selected assays and liver damage was evaluated by classifying responses as true positive (TP), true negative (TN), false positive (FP), or false negative (FN) for a *χ* *^2^* (α = 0.05) or correct classification rate (CCR) test. (*A*) The biological response profile (red = active or toxic, blue = inactive or non-toxic, yellow = inconclusive or untested) of liver damage compounds represented in the heat map using the top four assays (AIDs 686978, 743067, 743140, and 743202). Individual assays show weak IVIVC, but the combined responses of the assays using threshold RA > 0.25 as active resulted in a statistically significant IVIVC (*χ* *^2^* *p*-value = 0.000292). (*B*) The IVIVC between experimental quantitative high-throughput screening antioxidant respionse element *beta* lactamase reporter gene (qHTS ARE-*bla*) activation and liver damage and the QSAR predictions for each liver damage compound, for subsets of overlapping compounds with potential toxicophores A (left) and B (right).

*Liver toxicants profile and its IVIVCs.* The goal of the automatic data mining and extraction tool used in the present study was to reduce the big data pool to a much smaller size that could be manually curated by experts. The profiling tool identified 2,978 assays (available upon request from the corresponding author) relevant to qHTS ARE-*bla* activation and/or liver damage, 958 of which existed in both profiles. Automated data extraction identified 20 PubChem assays based on the first three criteria for assay selection (appeared in both profile groups, contained > 10 active responses that matched the inputted data, CCR > 0.5 and *L* ≥ 1). The assays are listed in Supplemental Material, Table S1. However, automatic methods cannot detect the detailed characteristics of an assay and distinguish the difference between *in vitro* and *in vivo* assays. The 20 assays identified by the initial automated screening procedure were manually reviewed to confirm that they met the *in vitro* selection criterion. For example, AID 1199 was identified as an *in vivo* assay; it did not fit the “*in vitro* assay” criterion and was removed. A total of 8 non–*in vitro* assays were removed in this step, and 12 *in vitro* assays remained. The literature search revealed no information to support the relevance of six assays (AIDs 121, 123, 589, 590, 2330, and 720532) to either liver damage or oxidative stress. Six assays remained, two of which had redundant activities. For example, AIDs 686978 and 686979 refer to the qHTS human tyrosyl-DNA phosphodiesterase 1 (TDP1) assay tested under two different conditions, and the activities for most of the compounds were the same. AID 686978 was selected because the assay was performed in the absence of the topoisomerase I poison camptothecin, which was more suitable for the present study. AIDs 743065 and 743067 are both qHTS assays to identify small-molecule antagonists of the thyroid receptor (TR) signaling pathway; AID 743067 was selected because it is a summary assay (i.e., it includes both primary and cell viability counter-screen results). After removing the redundant assays and evaluating the remaining assays by their mechanisms, four PubChem assays remained: AID 686978, qHTS for inhibitors of TDP1; AID 743067, qHTS assay to identify small-molecule antagonists of the TR signaling pathway; AID 743140, qHTS assay to identify small-molecule agonists of the peroxisome proliferator-activated receptor gamma (PPARγ) signaling pathway; and AID 743202, which was the qHTS ARE-*bla* assay used in the QSAR models described above. These assays are relevant to ARE perturbation and liver damage, according to the literature (Fielden et al. 2007; Königer et al. 2014; Malik and Hodgson 2002; Mantena et al. 2008), and were combined to create the biological response profile (Figure 3A).

Although these four assays met the selection criteria, the individual assay predictions were not significantly associated with *in vivo* liver damage (*χ^2^ p-*values for the independence of assays and *in vivo* liver damage = 0.24–0.59). However, combining these four assays and defining toxicity as RA > 0.25 resulted in a statistically significant association (*χ^2^ p-*value = 0.000292). The biological profile shows the responses for 953 compounds from the liver damage data set against the top four assays and their combined responses, using a threshold RA > 0.25 (Figure 3A). We note that 361 liver damage compounds are not shown because no bioassay data were available for them.

The qHTS ARE-*bla* data set used in this study contained > 6,000 compounds but does not cover all of the compounds in the liver damage data set. Therefore, qHTS ARE-*bla* combinatorial QSAR models were used to predict the activity of compounds that were not tested in the qHTS ARE-*bla* study. It is important to note that the liver damage data set consisted of mostly drug-like compounds that were outside the AD of the QSAR models. In previous studies, QSAR models typically could not predict compounds outside the AD as accurately as compounds within the AD (Tropsha and Golbraikh 2007). As shown in the principal component analysis (Figure 2B) and according to the AD analysis, most of the liver damage data set compounds either shared the same chemical space as the actives in the modeling set or were outside the AD, meaning they were likely to be predicted to be active by the QSAR models. This result led to an increase of false positives in the later IVIVC analysis, providing a hint that extra experimental ARE data are still needed for analyzing the drug-like compounds of interest in future studies.

CASE Ultra and ChemoTyper identified two subsets of compounds. The subsets contained a chemical fragment that showed a statistically significant IVIVC between ARE-*bla* activation and liver damage in the *χ^2^* test (*p*-value = 0.01) and are referred to as potential toxicophores A and B (Figure 3B). There were more true positives than false positives. Therefore, the active responses in this assay are potential signals of liver damage for the compounds that contain the potential toxicophores.

Furthermore, the qHTS ARE-*bla* combinatorial QSAR models were used to predict liver damage data set compounds without experimental qHTS ARE-*bla* perturbation results. Figure 3B shows the IVIVC (TP, TN, FP, and FN) between qHTS ARE-*bla* activation and liver damage for compounds with potential toxicophores A and B, using experimental ARE-*bla* data and QSAR predictions. When using only QSAR results, the IVIVC was not statistically significant (*χ^2^ p*-value = 0.41) for both potential toxicophores. This lack of significance is due to structural differences between the drugs in the liver damage data set and the compounds in the Tox21 data set used to develop the qHTS ARE-*bla* combinatorial QSAR model, as described above. The result suggests a limitation of applying QSAR models to predict new compounds that are outside of the AD.

## Discussion

ARE pathway perturbation is an important mechanism for alleviating and preventing oxidative stress (Ma 2013). In the present study, qHTS ARE-*bla* data and the resulting QSAR models were used to study the relationship between oxidative stress and liver damage. When qHTS ARE-*bla* data for a compound were not available, the combinatorial QSAR models were used to fill in the data gap. This technique can be adapted to populate response profiles for other assays.

**Figure 4:**
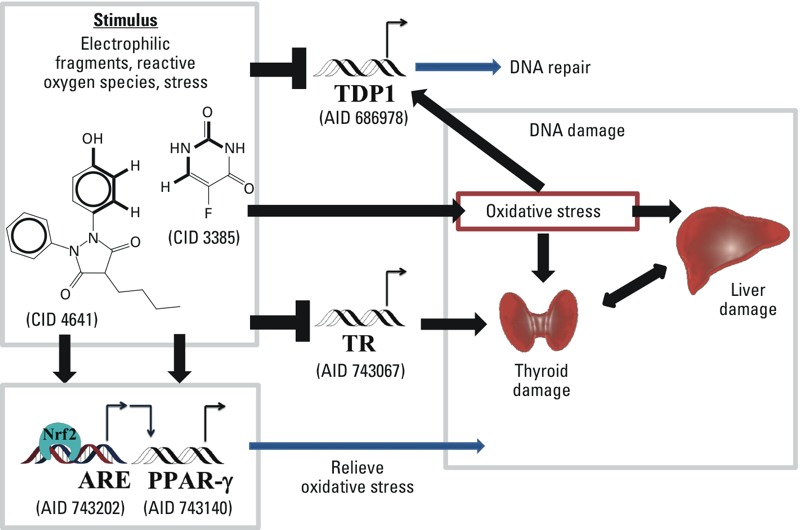
The potential liver toxicity mechanism of compounds such as oxyphenbutazone (CID 4641) and 5-fluorouracil (CID 3385), which contain either of the proposed toxicophores A or B, can generate reactive oxygen species. These types of stimuli activate the antioxidant response element signaling pathway (ARE) (AID 743202) and the peroxisome proliferator-activated receptor gamma (PPARγ) signaling pathway (AID 743140), inhibit the human tyrosyl-DNA phosphodiesterase 1 (TDP1) signaling pathway (686978), or disrupt the thyroid receptor (TR) signaling pathway (AID 743067).

The workflow created in this study used data from PubChem, a publicly available big data source, to create and populate a bioassay response profile that revealed the relationship between oxidative stress and liver damage (Figure 1). Furthermore, the workflow in this study can be adapted to develop adverse outcome pathways (AOPs) (Ankley et al. 2010). Our study identified a combination of molecular initiating events (MIEs) (Allen et al. 2014) between certain drugs and biomolecules that could cause adverse outcomes resulting in liver damage. Combinations of drugs or compounds (e.g., lipids) carrying fragments susceptible to free-radical oxidation and fragments that can inhibit signaling pathways meant to alleviate or prevent oxidative stress can all lead to liver damage. These MIEs and their adverse outcome(s) are described in the following paragraphs and are illustrated in Figure 4.

Assay AID 686978 identifies inhibitors of human tyrosyl-DNA phosphodiesterase 1 (TDP1). TDP1 is an enzyme that repairs single-stranded DNA breaks covalently linked to topoisomerase I. Mutations in TDP1 impair the ability of a cell to repair DNA damaged by oxidation or drugs (Ben Hassine and Arcangioli 2009). When DNA is damaged and TDP1 is inhibited, topoisomerase I stays covalently linked to the DNA during replication, and the cell dies (Pouliot et al. 1999). Because the ARE pathway contains a considerable number of detoxifying genes, it acts as the first line of defense to prevent DNA damage from oxidation or drugs (Kwak et al. 2003).

Active compounds in assay AID 743067 act as TR antagonists and can disrupt metabolic homeostasis by inhibiting the binding of the thyroid hormone (Jameson and Weetman 2012). The liver plays a major role in thyroid hormone metabolism, and liver damage is often associated with thyroid diseases (Huang and Liaw 1995). Furthermore, the liver metabolizes lipids, and thyroid hormones regulate hepatic lipid homeostasis (Malik and Hodgson 2002). Lipids autoxidize in the presence of molecular oxygen, a process known as lipid peroxidation (Porter et al. 1995), and form free radicals and ROS. Typically, the ARE will inactivate ROS (Shukla et al. 2012). Failure to terminate ROS results in oxidative stress (Sies 1997), particularly when a TR antagonist has disrupted liver lipid metabolism.

Assay AID 743140 identifies PPARγ agonists that activate the PPAR response elements and, in this specific case, regulate adipogenesis (Tontonoz et al. 1994). Adipose tissue, in particular visceral adipose tissue, releases fatty acids directly into the liver via the hepatic portal vein (Lafontan and Girard 2008). Fatty acids are susceptible to lipid peroxidation. Disrupting PPARγ and adipogenesis could put the liver at risk for oxidative stress when fatty acids are in excess.

The AOP concept has been presented as a logical sequence of biological responses that is useful for understanding complex toxicity phenomena (Allen et al. 2014; Ankley et al. 2010). Allen et al. (2014) discussed a unified MIE definition for the AOP framework based on the AOP concept for risk assessment purposes. This type of research uses *in vitro* methods to classify compounds by mode of action. Therefore, the chemical *in vitro–in vivo* relationships identified in this study can also be integrated into the AOP framework of liver damage. Potential toxicophore A is an electrophilic fragment that is highly susceptible to free radical oxidation owing to its allylic hydrogen (Porter et al. 1995). It represents a key chemical property of potential toxicants in an AOP framework. For example, oxyphenbutazone (CID 4641) is known to cause liver damage (Gaisford 1962). It contains potential toxicophore A and is active in AIDs 686978 and 743202 as a TDP1 inhibitor and an ARE agonist, respectively. The bioassay results can be viewed as the macromolecular interactions, and the RA value can be considered to be a specific cellular response pathway perturbation score (i.e., ARE signaling pathway perturbation and TDP1 inhibition) of the AOP for this compound. The molecular mechanism by which oxyphenbutazone causes liver damage remains unclear (Gaisford 1962; Tai 2012); however, it is well established that oxyphenbutazone is a lipid-soluble drug metabolized by liver microsomal enzymes and requires molecular oxygen to metabolize (Davies and Thorgeirsson 1971). Similarly, potential toxicophore B is known as *N*-methylformamide, a well-known liver toxicant susceptible to free radical oxidation by C-H abstraction from alkyl group(s) adjacent to the nitrogen atom (Borduas et al. 2015). This reaction produces methyl isocyanate, which is highly toxic (Varma 1987). For example, 5-fluorouracil (CID 3385) contains toxicophore B. 5-Fluorouracil was shown to be active in AIDs 686978 and 743067, TDP1 inhibition and TR antagonism, respectively. If administered orally, 5-fluorouracil is metabolically degraded predominantly in the liver by dihydropyrimidine dehydrogenase (DPD) (Omura 2003). Patients who lack DPD are highly likely to experience liver damage (Chabner et al. 2011). In the present study, it is notable that the four major components of an AOP (as defined by Ankley et al. 2010) are included: chemical properties of toxicants, macromolecular interactions, cellular responses, and organ responses. Subsequent studies will focus on the AOP framework of liver damage by differentiating the hepatotoxicity mechanisms of liver damage (e.g., acute hepatic failure, cytolytic hepatitis, hepatic necrosis) (Zhu and Kruhlak 2014).

Our findings suggest that the four identified assays (AIDs 686978, 743067, 743140, and 743202) could be used to screen for compounds that cause oxidative stress and induce liver damage. When specific chemical features (e.g., potential toxicophores A and B) are present, the active responses obtained from these bioassays suggest potential hepatotoxicity. Although the four assays cover several important mechanisms of oxidative stress, negative results from all four assays would not be sufficient to indicate that a chemical is not hepatotoxic. Additional work on this project will include validation of these assays for their predictivity of liver damage, which will be used to optimize predictive liver toxicity models.

## Conclusions

We developed a workflow that identified assays from a public big data source for the evaluation of liver damage caused by oxidative stress. Although using four assays will not be sufficient to cover all of the relevant toxicity mechanisms of liver damage, this work clearly indicates the benefits of searching for useful toxicity data on compounds of interest in the public big data domain. The increase in false positives in the IVIVC analysis indicates that bioassay data are still needed for compounds outside the AD (e.g., drug-like compounds). This issue could be resolved by rational design of the HTS chemical library that covers all of the relevant chemical space. New compounds containing the potential toxicophores can be tested using these four assays to assess potential liver damage caused by oxidative stress before proceeding to animal testing.

The workflow developed in this study can be easily adapted to study relationships between any bioassay and other *in vivo* exposure data to evaluate complex *in vitro–in vivo* relationships and to reveal toxicity mechanisms. Future directions of *in silico* modeling of animal toxicity induced by drugs and oxidative stress could include pharmacology studies.

## Supplemental Material

(359 KB) PDF
