# Supplementary material for: Mechanism Profiling of Hepatotoxicity Caused by Oxidative Stress Using Antioxidant Response Element Reporter Gene Assay Models and Big Data
Source: Environ Health Perspect. 2015 Sep 18;124(5):634–41. doi: 10.1289/ehp.1509763 (PMC4858396; doi:10.1289/ehp.1509763)
Supplement: (359 KB) PDF [file ehp.1509763.s001.acco.pdf]

Environ Health Perspect
DOI: 10.1289/ehp.1509763

Note to Readers: *EHP* strives to ensure that all journal content is accessible to all readers. However, some figures and Supplemental Material published in *EHP* articles may not conform to 508 standards due to the complexity of the information being presented. If you need assistance accessing journal content, please contact ehp508@niehs.nih.gov. Our staff will work with you to assess and meet your accessibility needs within 3 working days.

Supplemental Material

Mechanism Profiling of Hepatotoxicity Caused by Oxidative Stress Using the Antioxidant Response Element Reporter Gene Assay Models and Big Data

Marlene Thai Kim, Ruili Huang, Alexander Sedykh, Wenyi Wang, Menghang Xia, and Hao Zhu

Table of Contents

Table S1. Final 20 PubChem assays manually evaluated for consideration into the final biological response profile

Figure S1. Performance of all individual models and the consensus model in the 5-fold cross validation

Table S1. Final 20 PubChem assays manually evaluated for consideration into the final biological response profile

	Statistics for relationship to liver damage[a]								Statistics for relationship to ARE-*bla*[a]								
AID	TP[b]	FP	TN	FN	Spec	Sens	CCR[c]	L[c]	TP[b]	FP	TN	FN	Spec	Sens	CCR[c]	L[c]	Title[d]
121	11	2	20	52	0.91	0.17	0.54	1.28	11	2	15	46	0.88	0.19	0.54	1.09	NCI human tumor cell line growth inhibition assay. Data for the K-562 Leukemia cell line
123	11	2	20	52	0.91	0.17	0.54	1.28	11	2	15	46	0.88	0.19	0.54	1.09	NCI human tumor cell line growth inhibition assay. Data for the MOLT-4 Leukemia cell line
248	21	6	54	66	0.90	0.24	0.57	2.07	13	2	33	56	0.94	0.19	0.57	2.20	NCI In Vivo Anticancer Drug Screen. Data for tumor model L1210 Leukemia (intraperitoneal) in B6D2F1 (BDF1) mice
256	22	5	25	34	0.83	0.39	0.61	1.96	12	2	11	32	0.85	0.27	0.56	1.18	NCI In Vivo Anticancer Drug Screen. Data for tumor model L1210 Leukemia (intraperitoneal) in CD2F1 (CDF1) mice
328	23	3	12	14	0.80	0.62	0.71	2.33	15	2	10	19	0.83	0.44	0.64	1.76	NCI In Vivo Anticancer Drug Screen. Data for tumor model P388 Leukemia (intraperitoneal) in B6D2F1 (BDF1) mice
330	27	5	33	45	0.87	0.38	0.62	2.38	15	4	20	43	0.83	0.26	0.55	1.24	NCI In Vivo Anticancer Drug Screen. Data for tumor model P388 Leukemia (intraperitoneal) in CD2F1 (CDF1) mice
589	23	12	70	117	0.85	0.16	0.51	1.04	27	5	35	131	0.88	0.17	0.52	1.14	qHTS Assay for Spectroscopic Profiling in 4-MU Spectral Region
590	22	12	68	112	0.85	0.16	0.51	1.01	27	4	35	124	0.90	0.18	0.54	1.39	qHTS Assay for Spectroscopic Profiling in A350 Spectral Region
1189	28	8	17	31	0.68	0.47	0.58	1.32	48	10	16	44	0.62	0.52	0.57	1.23	DSSTox (CPDBAS) Carcinogenic Potency Database Summary SingleCellCall Results
1199	19	4	12	27	0.75	0.41	0.58	1.32	36	8	10	35	0.56	0.51	0.53	1.01	DSSTox (CPDBAS) Carcinogenic Potency Database Summary Mouse Bioassay Results
1205	21	4	14	26	0.78	0.45	0.61	1.61	39	9	11	34	0.55	0.53	0.54	1.07	DSSTox (CPDBAS) Carcinogenic Potency Database Summary MultiCellCall Results
1996	70	28	3	3	0.10	0.96	0.53	1.03	70	17	5	7	0.23	0.91	0.57	1.11	Aqueous Solubility from MLSMR Stock Solutions

2330	11	6	111	152	0.95	0.07	0.51	1.13	21	5	48	147	0.91	0.13	0.52	1.10	Luminescence Cell-Based Primary HTS to Identify Inhibitors of STK33
686978[e]	74	46	139	200	0.75	0.27	0.51	1.06	99	31	80	218	0.72	0.31	0.52	1.08	qHTS for Inhibitors of human tyrosyl-DNA phosphodiesterase 1 (TDP1): qHTS in cells in absence of CPT
686979	67	31	144	201	0.82	0.25	0.54	1.37	86	29	85	220	0.75	0.28	0.51	1.07	qHTS for Inhibitors of human tyrosyl-DNA phosphodiesterase 1 (TDP1): qHTS in cells in presence of CPT
720532	39	25	159	236	0.86	0.14	0.50	1.00	40	10	102	273	0.91	0.13	0.52	1.30	qHTS for Inhibitors of binding or entry into cells for Marburg Virus
743065	61	44	122	163	0.73	0.27	0.50	1.00	96	27	79	203	0.75	0.32	0.53	1.22	qHTS assay to identify small molecule antagonists of the thyroid receptor (TR) signaling pathway
743067[e]	18	10	117	162	0.92	0.10	0.51	1.15	29	8	76	197	0.90	0.13	0.52	1.20	qHTS assay to identify small molecule antagonists of the thyroid receptor (TR) signaling pathway: Summary[e]
743140[e]	18	3	147	196	0.98	0.08	0.53	3.15	17	3	94	256	0.97	0.06	0.52	1.51	qHTS assay to identify small molecule agonists of the peroxisome proliferator-activated receptor gamma (PPARg) signaling pathway: Summary[e]
743202[e]	20	10	88	142	0.90	0.12	0.51	1.10	26	5	56	174	0.92	0.13	0.52	1.32	qHTS assay for small molecule agonists of the antioxidant response element (ARE) signaling pathway[e]

Abbreviations: TP, true positive; TN, true negative; FP, false positive; FN, false negative; Sens, sensitivity; Spec, specificity; CCR, correct classification rate; L, likelihood parameter.

The first three criteria used to in the automated selection process were that the assay had to [a]appear in both profile groups, [b]contain > 10 active responses that matched the inputted data, and [c]correlation was better than random (CCR > 0.5 and L ≥ 1). [d]Manual evaluation was required to determine if it was an *in vitro* assay and relevant to liver toxicity or oxidative stress. [e]AIDs 686978, 743067, and 743140, 743202 sufficed the criteria described.

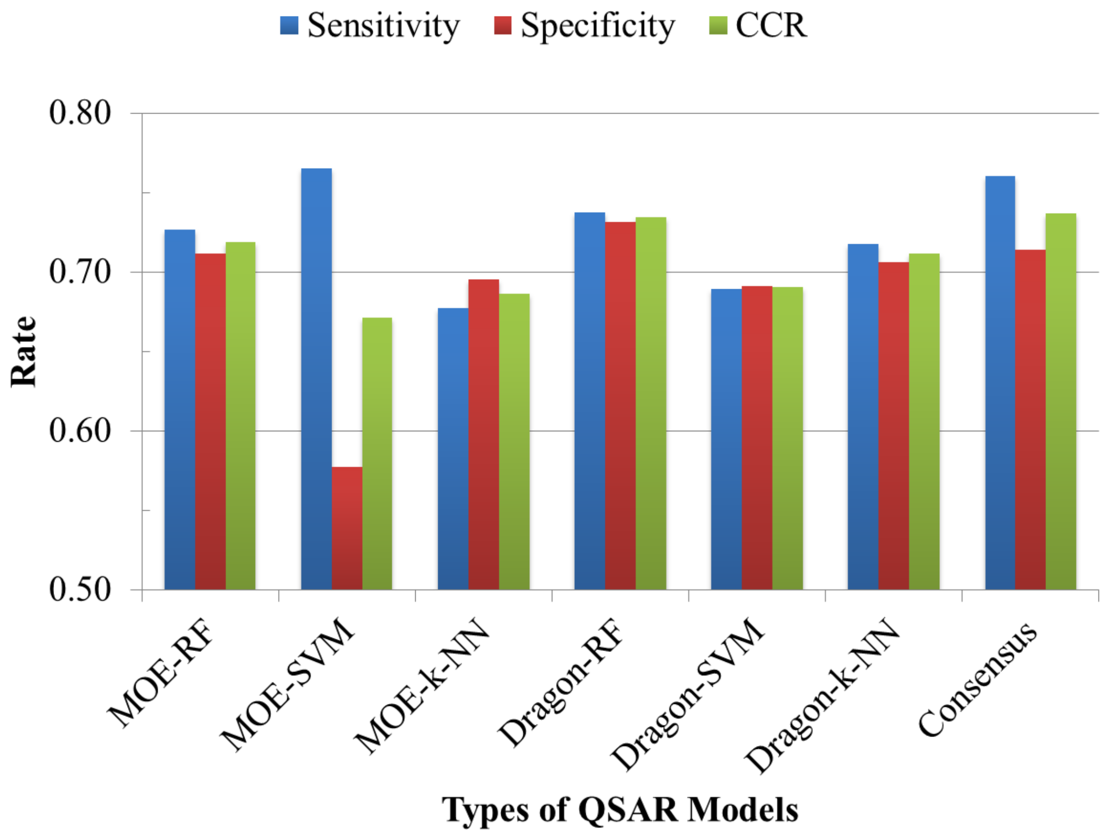

Figure S1. Performance of all individual models and the consensus model in the 5-fold cross validation
